# A Genome-Wide Association Study of Red Blood Cell Traits Using the Electronic Medical Record

**DOI:** 10.1371/journal.pone.0013011

**Published:** 2010-09-28

**Authors:** Iftikhar J. Kullo, Keyue Ding, Hayan Jouni, Carin Y. Smith, Christopher G. Chute

**Affiliations:** 1 Division of Cardiovascular Diseases, Mayo Clinic, Rochester, Minnesota, United States of America; 2 Biomedical Statistics and Informatics, Mayo Clinic, Rochester, Minnesota, United States of America; Universite de Montreal, Canada

## Abstract

**Background:**

The Electronic Medical Record (EMR) is a potential source for high throughput phenotyping to conduct genome-wide association studies (GWAS), including those of medically relevant quantitative traits. We describe use of the Mayo Clinic EMR to conduct a GWAS of red blood cell (RBC) traits in a cohort of patients with peripheral arterial disease (PAD) and controls without PAD.

**Methodology and Principal Findings:**

Results for hemoglobin level, hematocrit, RBC count, mean corpuscular volume, mean corpuscular hemoglobin, and mean corpuscular hemoglobin concentration were extracted from the EMR from January 1994 to September 2009. Out of 35,159 RBC trait values in 3,411 patients, we excluded 12,864 values in 1,165 patients that had been measured during hospitalization or in the setting of hematological disease, malignancy, or use of drugs that affect RBC traits, leaving a final genotyped sample of 3,012, 80% of whom had ≥2 measurements. The median of each RBC trait was used in the genetic analyses, which were conducted using an additive model that adjusted for age, sex, and PAD status. We identified four genomic loci that were associated (*P*<5×10^−8^) with one or more of the RBC traits (*HBLS1/MYB* on 6q23.3, *TMPRSS6* on 22q12.3, *HFE* on 6p22.1, and *SLC17A1* on 6p22.2). Three of these loci (*HBLS1/MYB*, *TMPRSS6*, and *HFE*) had been identified in recent GWAS and the allele frequencies, effect sizes, and the directions of effects of the replicated SNPs were similar to the prior studies.

**Conclusions:**

Our results demonstrate feasibility of using the EMR to conduct high throughput genomic studies of medically relevant quantitative traits.

## Introduction

As costs of genotyping continue to drop, accurate phenotyping is emerging as the rate-limiting step for conducting genomic studies. Consequently, there is considerable interest in leveraging the electronic medical record (EMR) for high-throughput phenotyping of diseases and medically relevant traits. Repositories of DNA from patients seen in the clinical setting can be matched with the EMR and genotyping/sequencing conducted to identify genetic variants associated with human diseases as well as related quantitative traits. Such an approach may reduce the time, effort, and cost involved in conducting genomic studies to identify disease susceptibility loci.

In 2007, National Human Genome Research Institute (NHGRI) funded the Electronic Medical Records and Genomics (eMERGE) consortium to develop and implement approaches for leveraging biorepositories with EMR systems for large-scale genomic research, including but not limited to genome-wide association studies (GWAS), sequencing, and structural variation [1]. The five participating sites include Group Health Cooperative − University of Washington, Marshfield Clinic, Mayo Clinic, Northwestern University, and Vanderbilt University. Each site chose to conduct a GWAS of a primary and supplementary phenotype. The Mayo Clinic proposal aims to identify genetic loci associated with peripheral arterial disease (PAD) and red blood cell (RBC) traits including hemoglobin, hematocrit, RBC count, mean corpuscular volume (MCV), mean corpuscular hemoglobin (MCH), and mean corpuscular hemoglobin concentration (MCHC).

Disorders involving RBCs, including anemia and polycythemia, have been associated with adverse cardiovascular outcomes as well as hypertension and heart failure [2], [3], [4], [5]. Prior studies indicate that RBC traits have a substantial genetic component with heritabilities of 0.56, 0.52, and 0.52 reported for RBC count, MCV, and MCH, respectively [6]. A genome-wide linkage scan in the Framingham Heart Study noted a significant linkage signal for RBC count (chromosomes 12p13 and 19p13), MCV (chromosome 11p15), and MCH (chromosome 11p15) [6]. Recently, the results of several GWAS for RBC traits in populations of European ancestry were reported [7], [8], [9], [10], with over 20 quantitative trait loci (QTL) identified. The objective of the present study was to assess the feasibility of leveraging the EMR to conduct a GWAS of quantitative traits, using RBC traits as an example. We investigated whether the QTL identified in recent GWAS of RBC traits [7], [8], [9], [10] could be replicated using trait values derived from the EMR. We first developed and validated an algorithm based on billing codes and natural language processing (NLP) of unstructured clinical notes, to exclude RBC trait values that may have been affected by comorbidities, marrow/immune suppressing medications, or major surgery. We then undertook a GWAS for RBC traits extracted from the Mayo Clinic EMR [11].

## Results

### Characteristics of participants

A total of 3,487 patients (PAD cases and controls), were recruited through 09/30/2009 for the Mayo Clinic eMERGE study. Figure S1A illustrates the process of extraction of RBC traits from the EMR. In total, 10 fields were extracted for each individual (Figure S1B). After using the unique test code for each RBC trait, as well as excluding RBC values obtained during hospitalization, 3,411 patients remained. Since the RBC traits are measured together as part of the complete blood count, the number of participants and laboratory tests were similar for six RBC traits and multiple measurements for each RBC trait were available in most individuals (Figure S1C).

### Assessment of comorbidities and medications that can affect RBC traits

**Figure 1:**
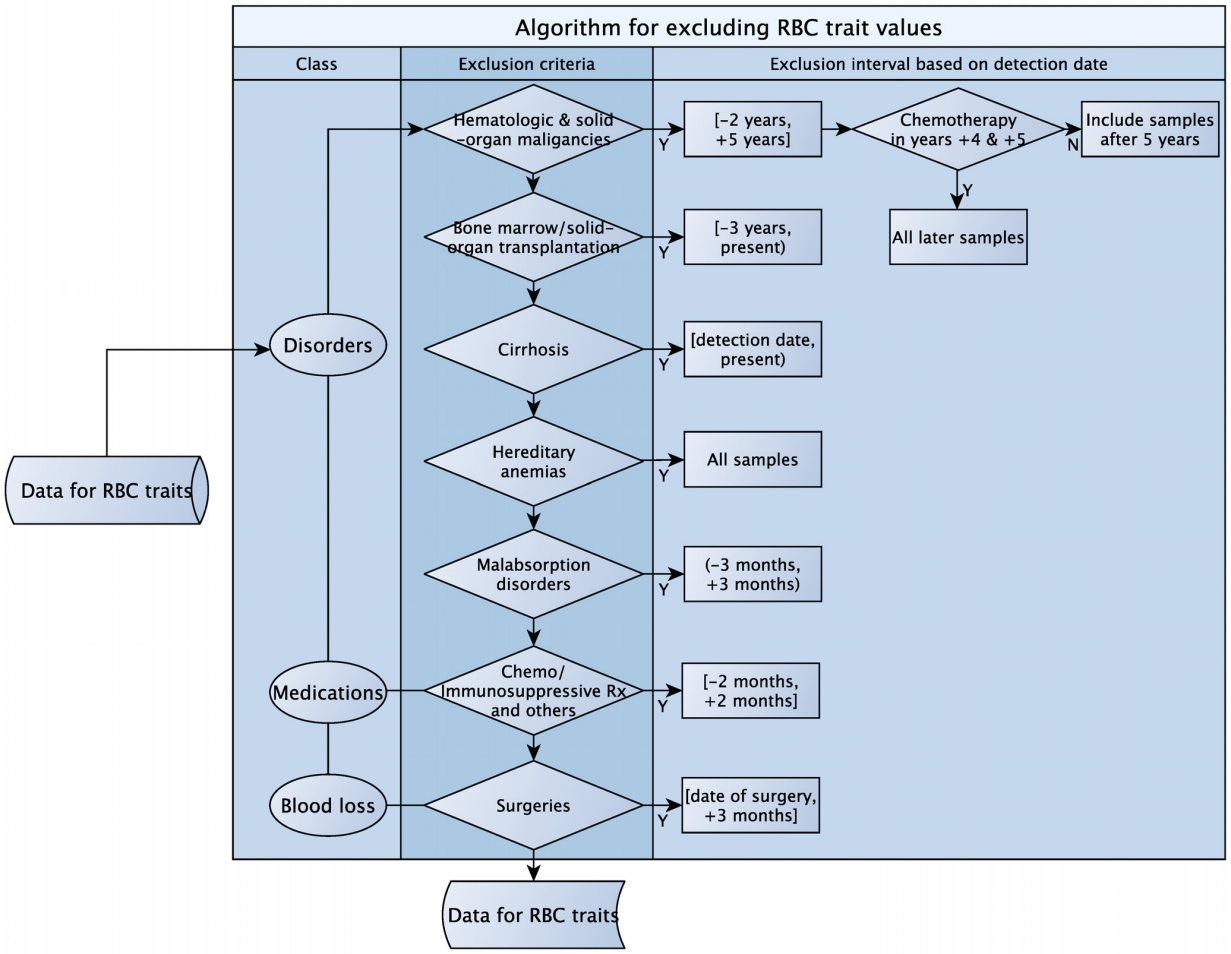
Algorithm for excluding RBC trait values affected by comorbidities, medications, and blood loss.

We excluded 12,864 records and 200 individuals based on the algorithm shown in Figure 1 and described in detail in Tables S1-S5. As a result, 3,012 genotyped patients with 20,650 values were included in the association analyses. We selected 50 sets of RBC trait values and performed a manual review of the EMR to assess whether any of the exclusion criteria were present at the time of the blood draw for these values. No exclusionary criteria were present at the time of the blood draws, thereby validating the algorithm. Characteristics for 3,012 individuals grouped by PAD status are summarized in Table 1.

**Table 1:** Sample characteristics.

	Total (*n* = 3,012)	PAD Cases (*n* = 1,478)	Controls (*n* = 1,534)	*P*
Age (years)	63.2±9.5	65.8±10.7	60.6±7.3	<0.0001
Men (%)	1868 (62.0)	951 (64.3)	917 (59.8)	0.01
Body mass index (kg/m^2^)	28.7±5.3	29.0±5.4	28.5±5.3	0.01
Hemoglobin (g/dL)	14.1±1.3 [13.3, 14.1, 15.0]	13.8±1.4 [13.0, 13.9, 14.8]	14.3±1.1 [13.5, 14.3, 15.1]	<0.0001
Hematocrit (%)	41.0±3.6 [38.7, 41.1, 43.5]	40.4±4.0 [37.9, 40.5, 43.1]	41.5±3.1 [39.3, 41.5, 43.7]	<0.0001
RBC count (×10^12^/L)	4.5±0.4 [4.3, 4.5, 4.8]	4.5±0.5 [4.2, 4.5, 4.8]	4.6±0.4 [4.3, 4,6, 4.9]	<0.0001
MCV (fL)	90.5±4.2 [87.9, 90.5, 93.0]	90.8±4.7 [87.9, 90.7, 93.6]	90.3±3.6 [88.0, 90.3, 92.6]	0.0007
MCH (pg)	31.1±1.6 [30.2, 31.1, 32.0]	31.1±1.8 [30.2, 31.1, 32.2]	31.1±1.4 [30.2, 31.1, 31.9]	0.36
MCHC (%)	34.3±0.5 [34.0, 34.3, 34.7]	34.3±0.5 [33.9, 34.3, 34.6]	34.4±0.5 [34.1, 34.4, 34.7]	<0.0001

Continuous variables are presented as mean ± SD; in addition, for the six RBC traits, the [25% quartile, median, 75% quartile] is also listed; categorical variables are presented as percentages (%).

### GWAS of RBC traits

**Figure 2:**
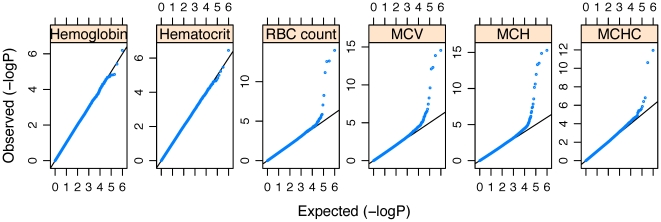
QQ plots for quantitative trait locus (QTL) analyses. The horizontal axis shows (–log_10_ transformed) expected *P* values, and the vertical axis indicates (–log_10_ transformed) observed *P* values.

**Figure 3:**
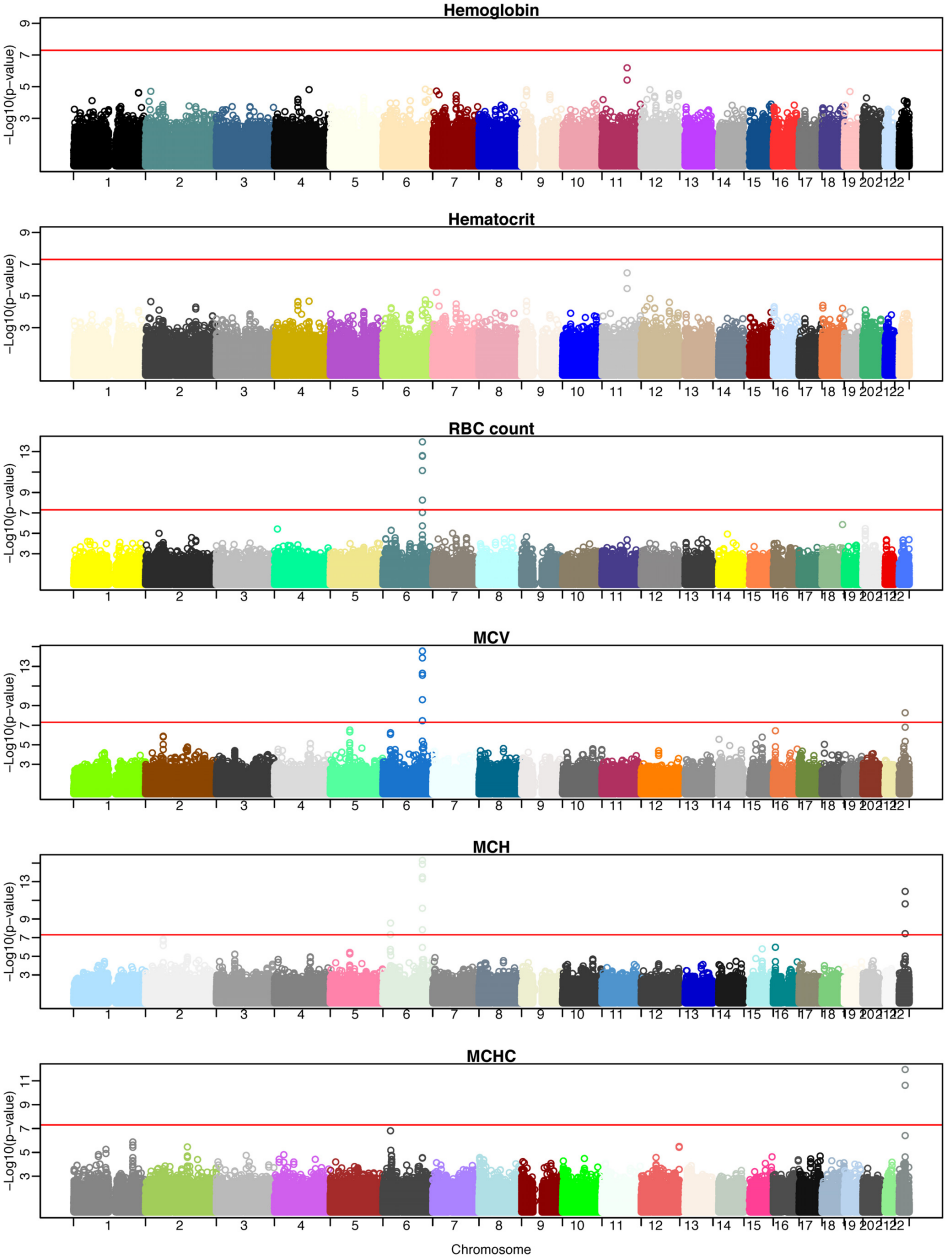
Manhattan plots for GWAS analyses of RBC traits. The vertical axis indicates (–log_10_ transformed) observed *P* values; and the horizontal line indicates the genome-wide significant level of *P* = 5×10^−8^.

The distribution of the number of measurements for each RBC trait is shown in Figure S2; ∼20.6% individuals had only one laboratory test and >95% had ≤20 laboratory tests. For individuals with multiple measurements, the median value was used in the analyses, which were performed under the additive model that adjusted for sex, age and PAD status, using *PLINK* [12]. We identified 11 significant SNPs (ie, *P*<5×10^−8^) within four genomic regions that were associated with four RBC traits. Quantile-quantile plots for the QTL for six RBC traits are shown in Figure 2, and Manhattan plots for the QTL are shown in Figure 3. Table 2 summarizes the chromosomal location, minor allele (minor allele frequency), effect size by the minor allele, variance explained by the associated loci, and *P* value for these SNPs. The variance of RBC traits explained by the associated SNPs ranged from 0.7%–2.2%.

**Table 2:** Genetic variants associated with RBC traits.

Trait	Chr	SNP	Position (bp)	Minor allele	MAF	Gene	*β* (SE)	R^2^ (%)	*P*
RBC count	6q23.3	rs7775698	135,460,328	T	0.26	*HBS1L/MYB*	−0.086±0.011	1.248	1.11E-14
RBC count	6q23.3	rs4895441*	135,468,266	G	0.27	*HBS1L/MYB*	−0.081±0.011	1.105	2.46E-13
RBC count	6q23.3	rs9376092	135,468,837	A	0.27	*HBS1L/MYB*	−0.081±0.011	1.102	2.85E-13
RBC count	6q23.3	rs9494145	135,474,245	C	0.24	*HBS1L/MYB*	−0.080±0.012	1.069	7.18E-12
RBC count	6q23.3	rs6569992	135,493,845	A	0.20	*HBS1L/MYB*	−0.073±0.012	0.709	5.57E-09
MCV	6q23.3	rs7775698	135,460,328	T	0.26	*HBS1L/MYB*	0.919±0.119	1.963	1.37E-14
MCV	6q23.3	rs4895441*	135,468,266	G	0.27	*HBS1L/MYB*	0.854±0.118	1.723	5.03E-13
MCV	6q23.3	rs9376092	135,468,837	A	0.27	*HBS1L/MYB*	0.846±0.118	1.695	7.94E-13
MCV	6q23.3	rs9494145	135,474,245	C	0.24	*HBS1L/MYB*	0.982±0.124	2.022	2.82E-15
MCV	6q23.3	rs6569992	135,493,845	A	0.20	*HBS1L/MYB*	0.844±0.133	1.285	2.50E-10
MCV	6q23.3	rs17064262	135,507,167	C	0.19	*HBS1L/MYB*	0.750±0.136	0.977	3.49E-08
MCV	22q12.3	rs855791	35,792,882	A	0.44	*TMPRSS6*	−0.620±0.106	1.011	5.41E-09
MCH	6q23.3	rs7775698	135,460,328	T	0.26	*HBS1L/MYB*	0.370±0.045	2.233	5.17E-16
MCH	6q23.3	rs4895441*	135,468,266	G	0.27	*HBS1L/MYB*	0.343±0.045	1.960	3.12E-14
MCH	6q23.3	rs9376092	135,468,837	A	0.27	*HBS1L/MYB*	0.340±0.045	1.931	4.94E-14
MCH	6q23.3	rs9494145	135,474,245	C	0.24	*HBS1L/MYB*	0.380±0.047	2.114	1.36E-15
MCH	6q23.3	rs6569992	135,493,845	A	0.20	*HBS1L/MYB*	0.333±0.051	1.413	7.05E-11
MCH	6q23.3	rs17064262	135,507,167	C	0.19	*HBS1L/MYB*	0.295±0.052	1.069	1.42E-08
MCH	6p22.2	rs17342717	25,929,749	T	0.10	*SLC17A1*	0.377±0.069	0.985	4.66E-08
MCH	6p22.1	rs1800562*	26,201,120	A	0.06	*HFE*	0.494±0.083	1.137	2.76E-09
MCH	22q12.3	rs855791	35,792,882	A	0.44	*TMPRSS6*	−0.289±0.040	1.525	1.10E-12
MCH	22q12.3	rs5756504	35,797,216	T	0.36	*TMPRSS6*	0.234±0.042	0.949	3.73E-08
MCH	22q12.3	rs4820268	35,799,537	G	0.47	*TMPRSS6*	−0.267±0.040	1.371	2.41E-11
MCHC	22q12.3	rs855791*	35,792,882	A	0.44	*TMPRSS6*	−0.084±0.013	1.345	2.40E-11
MCHC	22q12.3	rs4820268	35,799,537	G	0.47	*TMPRSS6*	−0.088±0.012	1.582	1.13E-12

The asterisk indicates the three replicated SNPs.

**Figure 4:**
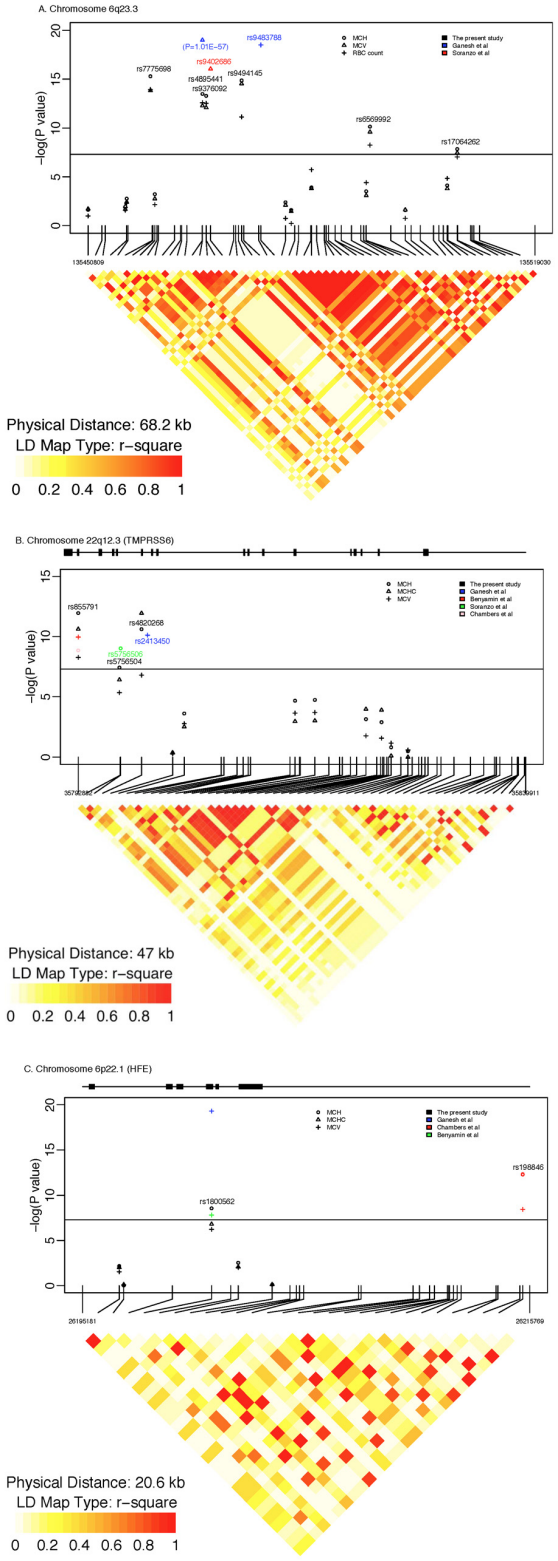
Regional plots of loci associated with RBC traits on chromosomes 6q23.3, 22q12.3, and 6p22.1. A. Regional plots of loci associated with RBC traits on chromosome 6q23.3: 135,450 kb – 135,520 kb. The top panel indicates the negative logarithm of observed *P* value, and the bottom panel indicates the patterns of LD based on HapMap (www.hapmap.org) CEU population. *HBS1L* (NM_006620) is located from bp 135,417,715 to 135,323,216; and *MYB* (NM_005375) is located from bp 135,544,146 to 135,582,002. B. Regional plots of loci associated with RBC traits on chromosome 22q12.3: 35,791 kb – 35,840 kb. *TMPRSS6* (NM_153609) is located from bp 35,791,425 to 35,829,639. Gene structure of *TMPRSS6* is shown. C. Regional plots of loci associated with RBC traits on chromosome 6p22.1: 26,195 kb – 26,216 kb. *HFE* (NM_000410) is located from bp 26,195,488 to 26,203,448. Gene structure of *HFE* is shown.

SNPs within the intergenic region of chromosome 6q23.3 [between HBS1-like (S. cerevisiae) (*HBS1L*) and v-myb myeloblastosis viral oncogene homolog (avian) (*MYB*)] were associated with RBC count, MCV, and MCH: five SNPs with RBC count (rs7775698 had the lowest *P-*value, *P* = 1.1×10^−14^, *R* ^2^ = 1.2%), six SNPs with MCV (rs9494145 had the lowest *P-*value, *P* = 2.8×10^−15^, *R* ^2^ = 2.0%) and MCH (rs7775698 had the lowest *P-*value, *P* = 5.7×10^−16^, *R* ^2^ = 2.2%). These SNPs were located within two different linkage disequilibrium (LD) blocks based on HapMap CEU samples (Figure 4A). SNPs rs7775698, rs4895441, rs9376092, and rs9494145 were located in the same LD block close to *HBS1L*, whereas rs6569992 and rs17064262 were close to *MYB*.

A nonsynonymous SNP (rs855791, V→A) within the transmembrane protease, serine 6 gene (*TMRPSS6*) on chromosome 22q12.3 was associated with MCV (*P* = 5.4×10^−9^, *R* ^2^ = 1.0%), MCH (*P* = 1.1×10^−12^, *R* ^2^ = 1.5%), and MCHC (*P* = 2.4×10^−11^, *R* ^2^ = 1.3%). We also noted other SNPs within this region to be associated with MCH [rs5756504 (intronic) and rs4820268 (synonymous)] and MCHC (rs4820268). These three SNPs showed a high level of LD (Figure 4B), suggesting that the nonsynonymous SNP (rs855791) is the likely causal locus.

A nonsynonymous SNP (rs1800562, C→Y) within the hemochromatosis gene (*HFE*) on chromosome 6p22.1 was associated with MCH (*P* = 2.8×10^−9^, *R* ^2^ = 1.1%) (Figure 4C). We also identified a novel locus, rs17342717 (intronic, *P* = 4.7×10^−8^, *R* ^2^ = 1.0%) that was associated with MCH, within solute carrier family 17 (sodium phosphate), member 1 (*SLC17A1*) gene on chromosome 6p22.2.

### Replication of significant loci identified in prior GWAS for RBC traits

We compared our results with recently reported GWAS of RBC traits in subjects of European ancestry [7], [8], [9], [10]. We were able to replicate three loci identified in these studies (Table 3). The minor allele frequencies in our study were similar to the HapMap CEU population. The direction of allele effects was consistent across the studies. Although the effect sizes (ie, regression coefficients) varied across different studies, the effect sizes in our study were similar to effect sizes in at least one of the prior studies. In order to compare the results among different studies, we plotted the distribution of *P* values and patterns of LD along these genomic regions (Figure 4).

**Table 3:** Comparison of the effect sizes of significant SNPs with those identified in previous GWAS for RBC traits.

Trait	Gene	Present study				Freq.^#^ (CEU)	Previous studies			
		SNP	Effect allele (freq)	Effect size (β)	*P*		SNP	Effect allele	Effect size (β)	Ref.
MCV	*HBS1L/MYB*	rs4895441	G (0.27)	0.854	5.03E-13	0.22	rs4895441	A	−0.008*	[7]
MCV	*HBS1L/MYB*	rs4895441	G (0.27)	0.854	5.03E-13	0.22	rs9402686	A	0.818	[8]
RBC count	*HBS1L/MYB*	rs4895441	G (0.27)	−0.081	2.46E-13	0.22	rs9483788	T	0.014	[7]
MCV	*TMPRSS6*	rs855791	A (0.44)	−0.620	5.41E-09	0.39	rs855791	A	−0.127	[10]
MCV	*TMPRSS6*	rs855791	A (0.44)	−0.620	5.41E-09	0.39	rs2413450	A	−0.005	[7]
MCH	*TMPRSS6*	rs855791	A (0.44)	−0.289	1.10E-12	0.39	rs855791	A	−0.330	[9]
MCH	*TMPRSS6*	rs855791	A (0.44)	−0.289	1.10E-12	0.39	rs5756506	C	0.137	[8]
MCV	*HFE*	rs1800562	A (0.06)	1.087	5.84E-07	0.04	rs1800562	A	0.222	[10]
MCV	*HFE*	rs1800562	A (0.06)	1.087	5.84E-07	0.04	rs1800562	A	0.012	[7]
MCV	*HFE*	rs1800562	A (0.06)	1.087	5.84E-07	0.04	rs1800562	A	1.408	[8]
MCV	*HFE*	rs1800562	A (0.06)	1.087	5.84E-07	0.04	rs198846	A	0.820	[9]
MCH	*HFE*	rs1800562	A (0.06)	0.494	2.76E-09	0.04	rs198846	A	0.370	[9]

*The direction of effect of rs4895441 in Ganesh et al. [7] is based on the major allele (A) instead of the minor allele (G).

#: Frequency of effect allele (the present study) in the HapMap CEU population.

The SNP rs4895441 within *HBS1L/MYB* (chromosome 6q23.3) has been found to be associated with MCV [7], and the SNP rs9402686 [in high LD with rs4895441 (HapMap CEU *r* ^2^ = 0.953)] identified by Soranzo et al. [8] was also associated with MCV. The SNP rs9483788 (*r* ^2^ = 0.602 with rs4895441) within this genomic region was associated with RBC count [7]. These SNPs seem to be located within an LD block (Figure 4A), close to *HBS1L*. In addition, we found this locus to be associated with MCH (*P* = 3.1×10^−14^), a finding not observed in previous studies.

The SNP rs855791 within *TMPRSS6* (chromosomal 22q12.1) was found to be associated with hemoglobin [7], [9], MCV [10], and MCH [9] in prior studies; it was associated with MCV (*P* = 5.4×10^−9^), MCH (*P* = 2.8×10^−9^), and MCHC (*P* = 2.4×10^−11^) in the present study (Figure 4B). SNPs rs2413450 (*r* ^2^ = 0.737 with rs855791) and rs5756506 (*r* ^2^ = 0.347 with rs855791) were also noted to be associated with MCV [7] and MCH [8], respectively. These SNPs showed a high level of LD.

The SNP rs1800562 within *HFE* (chromosomal 6p22.1) was previously identified to be associated with hemoglobin [7], hematocrit [7], and MCV [7], [8], [10]. The locus was associated with MCV (*P* = 5.8×10^−7^), MCH (*P* = 2.8×10^−9^), and MCHC (*P* = 1.5×10^−7^) in the present study. Chambers et al. [9] noted that SNP rs198846, located in a different LD block with rs1800562, was associated with MCV and MCH (Figure 4C).

## Discussion

The EMR contains diverse and rich phenotypic information and DNA repositories linked to the EMR allow rapid assembly of patient sets for genomic studies. However, the utility of EMR-based approaches for discovery or validation of genotype-phenotype associations remains unproven. In the present study, we demonstrate that a biorepository matched to the EMR can be leveraged to conduct a GWAS of RBC traits. We extracted RBC traits values over a span of 15 years from the EMR, and used a billing code and NLP-based algorithm to exclude values that may have been affected by comorbidity, medication use or major surgery. We identified 11 unique significant SNPs (*P*<5×10^−8^) within four genomic loci associated with four RBC traits. Of these, three genomic loci (ie, *HBS1L/MYB*, *TMPRSS6*, and *HFE*) recently identified as being associated with RBC traits, were replicated, highlighting that phenotypes extracted from the EMR can be used for GWAS of quantitative traits. The fourth genomic locus − *SLC17A1* − a gene involved in sodium-phosphate co-transport system in the kidney, is a novel locus that we found to be associated with MCH.

Application of the GWAS approach to quantitative traits obtained from the EMR presents several challenges [1], [13]. Data integration from the EMR often requires querying across different data sources using different information extraction procedures [14]. In the present study, we used several separate data sources across the Mayo EMR (Figure S1A) to ensure the accuracy and completeness of the RBC trait values, making it feasible to conduct the GWAS. An additional challenge in using the EMR for genomic studies is assessment of comorbidities and medications that can affect the trait of interest. We used an algorithm that combined billing codes to identify comorbidities, procedure codes to identify surgeries associated with blood loss, and NLP to identify relevant medications, while retaining a sufficiently large sample size (Figure 1). We defined a time interval based on the detection date of the corresponding codes and excluded RBC trait values measured within this interval. Out of 35,159 RBC trait values in 3,411 patients, we excluded 12,864 values in 1,165 patients. However, since multiple tests for RBC traits were available in the EMR, this resulted in the exclusion of only 200 patients from the original sample.

A remarkable aspect of our study is that we were able to identify 11 SNPs in 4 loci influencing RBC traits at a genome-wide significance level using EMR-derived phenotypic data in only 3,012 patients. In spite of comorbidities such as chronic kidney disease and chronic obstructive lung disease that can affect RBC traits in PAD patients, we were able to replicate loci associated with RBC traits in prior cohort studies. Three of the four loci had been recently identified in GWAS that included much larger numbers of participants. Although we did not replicate all genomic loci from these prior studies, the loci we detected are the only ones that were found in at least two previous studies. Our findings are encouraging from the viewpoint of using the EMR for genomic studies. When compared with the previous studies for RBC traits [7], [8], [9], [10], the directions of effect alleles were the same and the effect sizes of the alleles were comparable to our study (Table 3). The variance explained by the associated loci ranged from ∼1%–2%, similar to the prior studies.

The molecular functions of the four genomic regions that were associated with RBC traits are summarized in Table 4. In addition to regulating fetal globin expression [15], *HBS1L/MYB* may have additional roles in erythropoiesis [16]. TMPRSS6 is a type II membrane-anchored serine protease that is involved in matrix remodeling processes in the liver [17], and is essential for normal iron homeostasis [18]. HFE and transferrin directly compete for binding to the transferrin receptor, thereby lowering its affinity for iron-containing transferrin and down-regulating uptake of iron by cells [19]. SLC17A1 plays an important role in phosphate homeostasis in animals and humans; how variants in this gene might influence MCH needs further investigation [20]. Of note, an intronic SNP rs17270561 (HapMap CEU *r* ^2^ = 0.51 with rs17342717) within *SLC17A1* was found to be associated with transferrin saturation (*P* = 5×10^−8^), by Benyamin et al [21].

**Table 4:** Molecular function of associated loci.

Symbol	Chr.	Gene	Function	Ref.
*HBS1L/MYB*	6q23.3	HBS1-like/v-myb myeloblastosis viral oncogene homolog (avian)	Regulates fetal globin expression	[15]
*TMPRSS6*	22q12.3	transmembrane protease, serine 6	Acts by cleaving hemojuvelin*	[26]
*HFE*	6p22.1	hemochromatosis protein isoform 1 precursor	Binds tightly to transferrin receptor 1, and reduces binding of transferring	[19]
*SLC17A1*	6p22.2	solute carrier family 17 (sodium phosphate), member 1	Essential for phosphate homeostasis in animals and humans	[20]

*hemojuvelin is essential for production of the iron regulatory hormone hepcidin [27].

### Limitations

A limitation of the use of EMR in genomic studies is the potential for selection and referral bias. Considerable effort may be needed to develop and validate phenotyping algorithms. The present study required a combined approach of NLP to identify prescribed medications and billing codes to exclude RBC values that might have been affected by chronic disease or medication use, while capturing a sufficiently large sample size. How well the genetic architecture of quantitative traits can be delineated from EMR-based genomic studies may vary with the trait of interest and will be influenced by trait heritability, variance in trait values, and how comorbidities affect trait values. In the present study, our ability to replicate may have been made easier by the fact that measurement of RBC traits is relatively precise in the clinical setting, trait values are stable over times, values may be relatively less affected by acute phase response, and that the traits have relatively high heritability. Additional GWAS of several quantitative traits are currently in progress within the eMERGE consortium, and will provide further insights in this regard.

### Future directions

The present study lays the groundwork for a GWAS of RBC traits across the five eMERGE sites (n = ∼17,000). We anticipate detection of additional novel genetic loci influencing RBC traits in the consortium-wide analyses. Although the availability of multiple measurements of a trait within the EMR may provide a more precise estimate of the trait value as well as change in trait value over time, it is not clear how to deal with multiple measurements in GWAS analyses. We are investigating the statistical power of different regression methods in dealing with multiple measurements. Finally, consistent with the goals of the eMERGE network, we are developing phenotyping algorithms to enable EMR-based genomic studies of other medically relevant quantitative traits and assessing the extent to which the algorithms are portable across EMR systems.

In conclusion, we demonstrate the use of the EMR to replicate genetic loci associated with inter-individual variation in RBC traits in prior cohort studies. As genotyping costs continue to decrease, phenotyping is emerging as the major bottleneck for identifying genetic loci influencing disease susceptibility or variation in medically relevant quantitative traits. Mining of the EMR is a high throughput, relatively inexpensive method to facilitate genetic studies of quantitative traits. Increasing use of the EMR affords an opportunity to expedite the investigation of genetic architecture of common and rare diseases as well as quantitative traits of medical importance.

## Materials and Methods

### Study participants

In October 2006, a biorepository of plasma and DNA samples was initiated by recruiting patients referred for lower extremity arterial evaluation to the Mayo Clinic's non-invasive vascular laboratory and individuals referred to the stress ECG laboratory to screen for coronary artery disease. Between October 2006 and May 2009, 3,527 patients were recruited. We used the following criteria to define presence of PAD: 1) an ankle brachial index (ABI) ≤0.9 at rest or 1 min after exercise; or 2) presence of poorly compressible arteries; or 3) normal ABI but prior history of revascularization for PAD [22]. All participants gave their written informed consent for participation in the study and the use of their data for future research. The study protocol was approved by the Institutional Review Board of the Mayo Clinic. The Mayo EMR began accumulating data in the early 1990s [23] and now includes all inpatient and outpatient billing codes, laboratory values, reports, and clinical documentation, almost all in electronic formats available for searching [11]. It currently contains over 120 million documents on ∼2 million patients. Patient-level data elements in the Mayo EMR included demographics, outpatient visits and hospitalizations, providers, diagnosis and procedure codes, and RBC trait values. Birth date, race, sex, ethnicity were obtained from the demographic database; the categories for race were ‘White,’ ‘Black or African American,’ ‘Hispanic,’ ‘Asian/Pacific Islander,’ ‘American Indian/Alaskan Native,’ ‘Others,’ ‘Unknown,’ and ‘Choose not to disclose.’

### RBC traits

The complete blood count is a commonly performed laboratory test [24] and includes the following RBC traits: (1) hemoglobin level: the concentration of hemoglobin within whole blood; (2) hematocrit, the percentage of whole blood comprising cellular erythrocyte elements; (3) RBC count, the number of red blood cells per volume of blood; (4) mean corpuscular volume (MCV), the average erythrocyte volume; (5) mean corpuscular hemoglobin (MCH), the average mass of hemoglobin per RBC in a sample of blood; and (6) mean corpuscular hemoglobin concentration (MCHC), the concentration of hemoglobin in a given volume of packed RBC.

### Data integration from the EMR

To extract data for RBC traits, we used separate relational databases as well as semi-structured data sources in the Mayo EMR. A schematic depicting extraction of RBC traits from the EMR is shown in Figure S1A. The data extracted for the period 01/01/1994 to 09/30/2009 included the test code and description, date and time of sample, units of results, associated reference range and indicators for low/high results, lab accession number, and results of the test in both character and numeric format (Figure S1B). Any RBC trait values obtained during an inpatient hospitalization (admit date≤sample date≤discharge date) were excluded unless these were only tests available for a patient.

### Assessment of comorbidities and medications that can affect RBC traits

Since RBC traits are affected by a wide array of medical conditions, we developed an EMR-based algorithm that includes billing codes and NLP of unstructured clinical notes to exclude values affected by comorbidities, medications or blood loss (Figure 1, and Tables S1-S5 and Methods S1). We compiled the International Classification of Disease 9 Clinical Management (ICD-9 CM), procedural ICD-9, and Current Procedural Terminology (CPT-4) codes indicative of clinical conditions that may affect RBC traits. The medical conditions included hematologic and solid-organ malignancies, bone marrow and solid-organ transplantation, cirrhosis, hereditary anemias, and malabsorption disorders. The medications included chemotherapeutic and immunosuppressive drugs. The algorithm is described in detail in the supplementary materials. Out of 35,159 RBC trait values in 3,411 patients, we excluded 12,864 values (in 1,165 patients) that had been measured during hospitalization or in the setting of hematological disease, malignancy, or use of drugs that affect RBC traits. As a result, 200 patients were excluded from the analyses.

### Association analyses

We used the median of a trait value when multiple results were available. Genotyping was performed at the Center for Genotyping and Analysis at the Broad Institute, using the Illumina Human660W-Quadv1_A genotyping platform, consisting of 561,490 SNPs and 95,876 intensity-only probes. Data were cleaned using the quality control (QC) pipeline developed by the eMERGE Genomics Working Group. This process includes evaluation of sample and marker call rate, gender mismatch and anomalies, duplicate and HapMap concordance, batch effects, Hardy-Weinberg equilibrium, sample relatedness, and population stratification. A total of 489,421 SNPs were used for analysis based on the following QC criteria: SNP call rate >98%, sample call rate >98%, minor allele frequency >0.05, Hardy-Weinberg equilibrium >0.001, 99.99% concordance rate in duplicates, and unrelated samples only. We excluded 11 samples with labeling errors. The data from all the patients, in addition to the HapMap III populations, were evaluated for population structure/substructure using *EIGENSTRAT* software [25], and those who were not in the European cluster were excluded (*n* = 42). After QC steps, 3,012 samples with phenotype and genotype data were available for association analyses (Figure S3).

Single-locus tests of association were performed in *PLINK* using linear regression analysis that assumed an additive genetic model and incorporated age, sex, and PAD case-control status as covariates [12]. To assess population structure, we examined the genomic control inflation factor (λ_GC_) for six RBC traits, and found these values to be below 1.020 without systematic inflation: 1.014 (hemoglobin), 1.017 (hematocrit), 1.007 (RBC count), 1.007 (MCV), 1.004 (MCH), and 1.016 (MCHC). After correcting for population structure using λ_GC_, the significant loci identified in the present study remained at *P*<5×10^−8^. The power of our study was ∼85% to detect a QTL that explains 1.5% variance in an RBC trait, given a sample size of 3,000, a minor allele frequency of 0.05, and the significance level of 5×10^−8^. The data for the consortium-wide analyses of RBC indices will be uploaded to dbGAP (www.ncbi.nlm.nih.gov/gap).

## Supporting Information

Figure S1 A. Schematic diagram of extracting data of RBC parameters from the EMR. B. The structure of extraction data from the EMR. Test description is the six RBC traits. C. Summary of the RBC traits in the extraction data. MCV, mean corpuscular volume; MCH, mean corpuscular hemoglobin; MCHC, mean corpuscular hemoglobin concentration.
(2.56 MB TIF)

Figure S2 Bar chart of the number of laboratory tests for RBC traits. MCV, mean corpuscular volume; MCH, mean corpuscular hemoglobin; MCHC, mean corpuscular hemoglobin concentration.
(0.24 MB TIF)

Figure S3 A flow chart of quality control of phenotypic and genotypic data for RBC traits in GWA studies.
(2.30 MB TIF)

Methods S1 Assessment of comorbidities and medications that can affect RBC traits.
(0.04 MB DOC)

Table S1 ICD-9-CM codes indicating the most commonly disorders that may affect RBC traits.
(0.09 MB DOC)

Table S2 ICD-9 and CPT-4 procedural codes indicating bone marrow and/or solid organ transplantation.
(0.05 MB DOC)

Table S3 CPT-4 codes indicating medications.
(0.04 MB DOC)

Table S4 Generic and brand names of commonly used oral chemotherapeutic and immunosuppressive medications.
(0.05 MB DOC)

Table S5 CPT-4 codes indicating anesthesia codes for surgeries that are likely to be associated with major blood loss and post-operative anemia.
(0.09 MB DOC)
