# Supplementary figures and images for: A Genome-Wide Association Study of Red Blood Cell Traits Using the Electronic Medical Record
Source: PLoS One. 2010 Sep 28;5(9):e13011. doi: 10.1371/journal.pone.0013011 (PMC2946914; doi:10.1371/journal.pone.0013011)

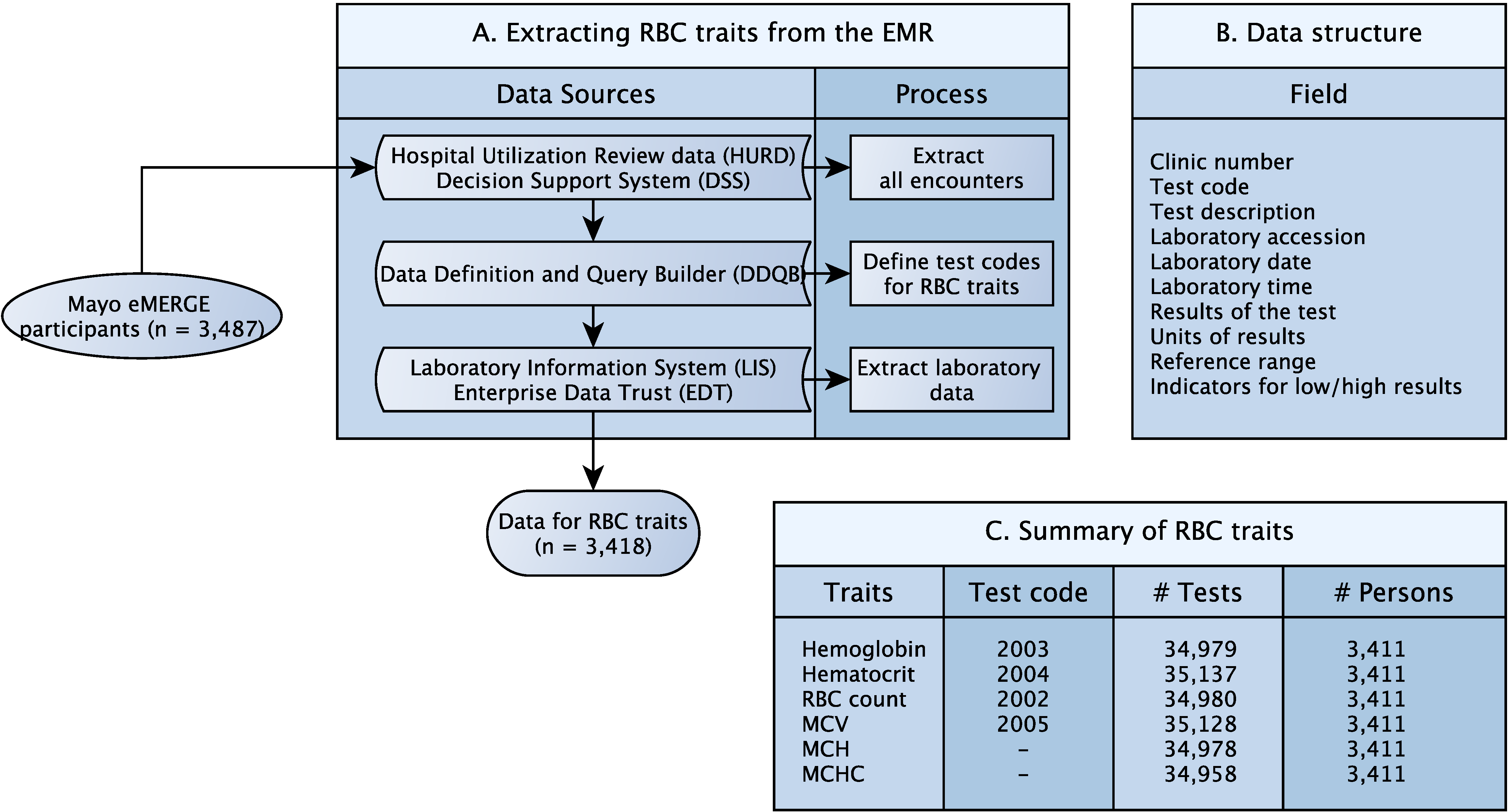

Supplement: Figure S1 — A. Schematic diagram of extracting data of RBC parameters from the EMR. B. The structure of extraction data from the EMR. Test description is the six RBC traits. C. Summary of the RBC traits in the extraction data. MCV, mean corpuscular volume; MCH, mean corpuscular hemoglobin; MCHC, mean corpuscular hemoglobin concentration. (2.56 MB TIF) [file pone.0013011.s001.tif]

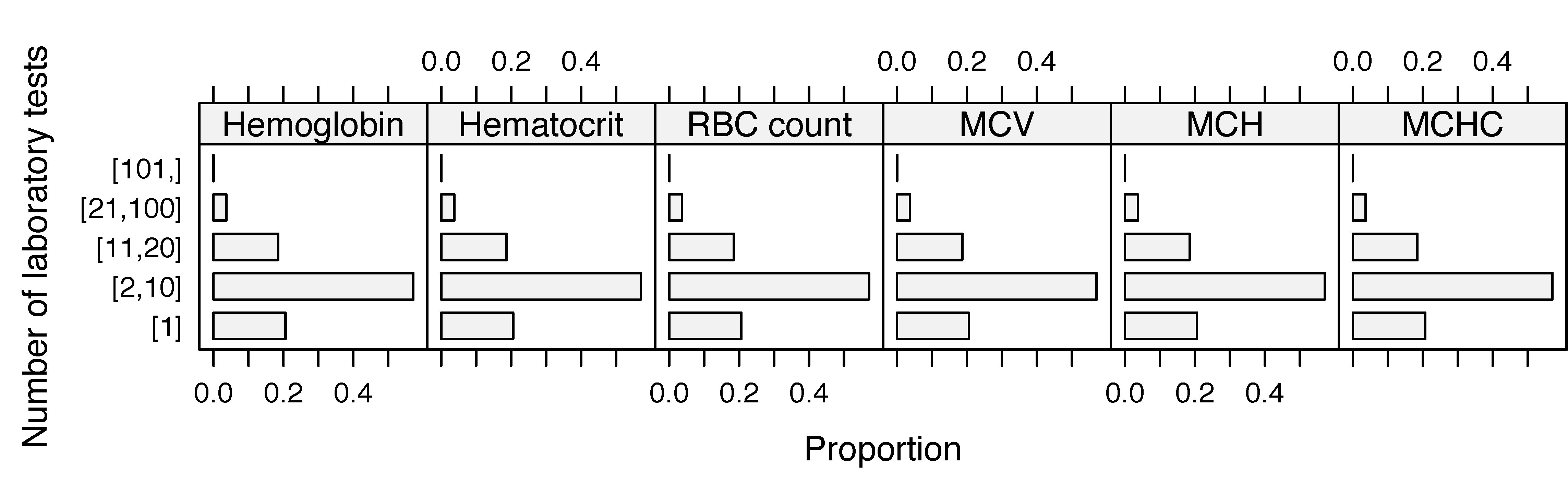

Supplement: Figure S2 — Bar chart of the number of laboratory tests for RBC traits. MCV, mean corpuscular volume; MCH, mean corpuscular hemoglobin; MCHC, mean corpuscular hemoglobin concentration. (0.24 MB TIF) [file pone.0013011.s002.tif]

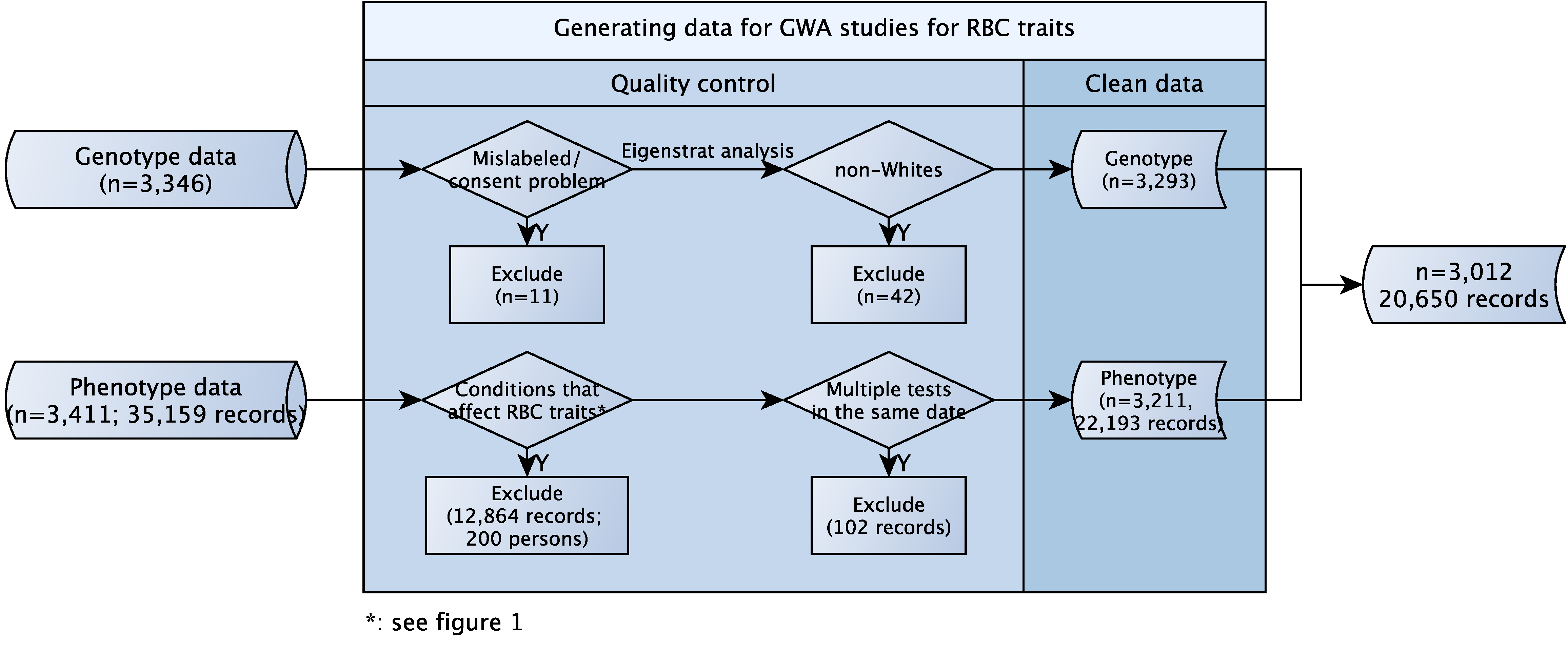

Supplement: Figure S3 — A flow chart of quality control of phenotypic and genotypic data for RBC traits in GWA studies. (2.30 MB TIF) [file pone.0013011.s003.tif]
