# Supplementary material for: A Genome-Wide Association Study of Red Blood Cell Traits Using the Electronic Medical Record
Source: PLoS One. 2010 Sep 28;5(9):e13011. doi: 10.1371/journal.pone.0013011 (PMC2946914; doi:10.1371/journal.pone.0013011)
Supplement: Methods S1 — Assessment of comorbidities and medications that can affect RBC traits. (0.04 MB DOC) [file pone.0013011.s004.doc]

**Assessment of comorbidities and medications that can affect RBC traits**

**List of comorbidities and medications that can affect RBC traits**

Since RBC traits are affected by a wide array of medical conditions and therapeutic interventions, several steps were taken in order to refine the pool of collected laboratory test results. The International classification of Disease 9 Clinical Management (ICD-9 CM), procedural ICD-9, and Current Procedural Terminology (CPT-4) coding systems were thoroughly reviewed for medical disorders and therapeutic medications/procedures that may affect RBC traits.

*Medical disorders affecting RBC traits*

We extracted the codes that correspond to anemias, hematologic/solid organ malignancies, spleen disorders, chronic kidney disease, cirrhosis, inflammatory bowel disease, malabsorption syndromes, autoimmune/connective tissue disease, organ transplantation, and other infectious diseases. An exhaustive list of the populated conditions and corresponding ICD-9/CPT-4 codes are listed in **Tables S1 and S2**.

*Medications*

In order to further enhance the detection of patients whose RBC results were not suitable for our analyses, CPT-4 and ICD-9 procedural codes were reviewed for codes representative of parenteral chemotherapy. These codes include ICD-9 procedural code (99.25, which covers all infusions of chemotherapeutic agents) while other CPT-4 codes are listed in **Table S3**. For orally administered chemotherapeutic and immunosuppressive medications, we populated a list of the commonly used medications using both generic and brand names. We then used NLP techniques to screen the EMR of study participants for the use of these oral medications. A detailed list of these oral medications can be found in **Table S4**.

*Blood loss*

The CPT-4 codes represent anesthesia codes for surgeries that are likely to be associated with major blood loss and post-operative anemia (**Table S5**).

***Exclusion Process***

The extracted ICD-9 CM, procedural ICD-9, and CPT-4 codes were used to screen our study participants for the presence of any of the previously mentioned conditions that may affect RBC indices. Corresponding codes were compiled for each person from the Mayo Clinic’s Institutional Planning Database (IPD), Hospital Utilization Review Database (HURD), and the Decision Support System (DSS). We aimed to exclude laboratory tests that were collected at times of active disorders affecting RBC indices. The algorithm for excluding RBC trait values is depicted in Fig. 1.

Samples from patients with hematologic and solid-organ malignancies were excluded if they were collected in the time period starting 2 years before and up to 5 years after the date of the first corresponding ICD-9 CM code. Afterwards, the electronic medical record for patients with malignancies was reviewed for the administration of chemotherapy starting at 3 years after the first ICD-9 CM that corresponds to the malignancy. Procedural ICD-9, CPT-4, and NLP for oral medications were used to that regard. If such use was detected, all later records were excluded. If no chemotherapy was administered during the aforementioned period of time, the patient will be likely cured and later records were included in the analyses.

Blood samples from patients who underwent bone marrow or solid organ transplantation were excluded if they were collected after the transplantation. Blood samples taken up to 3 years prior to transplantation were also excluded as they are probably not representative of the patient’s hematologic baseline. Test results for patients with cirrhosis were excluded starting from the earliest ICD-9 CM code representative of cirrhosis. All laboratory results belonging to patients with hereditary anemias such as sickle cell anemia, thalassemias, hemoglobin C and H diseases were excluded.

We then used the oral medications listed in Table S4 to screen our study participants for the use of medications that can potentially affect RBC indices (mainly taken for autoimmune/connective tissue disorders). NLP was implemented to that regard. Samples collected from patients taking these medications were excluded if they fell in a 4-month period centered upon the date at which such use was detected in the EMR.

Laboratory results for patients who underwent major surgeries (as evident through corresponding general anesthesia CPT-4 codes) were excluded if the samples were collected in the postoperative period and up to 3 months after the date of the CPT-4 code.

After applying the aforementioned general exclusion rules, the remaining samples were used for RBC indices analyses. Samples collected from patients with chronic kidney disease were not excluded and their test results were adjusted according to the patient’s renal function. Samples from patients with anemia, spleen disorders, anti-convulsant use (phenytoin and valproic acid), inflammatory bowel disease, chronic infectious diseases, and other autoimmune/connective tissue diseases were flagged to make them more accessible for later subgroup analyses.
