# Supplementary material for: A Genome-Wide Association Study of Red Blood Cell Traits Using the Electronic Medical Record
Source: PLoS One. 2010 Sep 28;5(9):e13011. doi: 10.1371/journal.pone.0013011 (PMC2946914; doi:10.1371/journal.pone.0013011)
Supplement: Table S1 — ICD-9-CM codes indicating the most commonly disorders that may affect RBC traits. (0.09 MB DOC) [file pone.0013011.s005.doc]

| **Table S1.** ICD-9-CM codes indicating the most commonly disorders that may affect RBC traits | |
| --- | --- |
| **Diseases** | **ICD-9 CM Code** |
| ***S1.1. Non-Malignant Hematologic Disorders*** | |
| Anemia | 280.9  281.1  281.3  281.4  281.9  282.2  282.9  284.9  285.9  336.2 |
| Iron deficiency anemia | 280.0  280.1  280.8  280.9  281.8 |
| Pernicious anemia  Vitamin B12 deficiency  Folic acid deficiency | 281.0  281.1  281.2  281.3  281.9  282.2  648.2 |
| Hemolytic anemia | 283.0  283.19  283.10  283.2  282.3  283.9 |
| Hemolytic uremic syndrome | 283.11 |
| Anemia of chronic disease | 285.29 |
| Malignancy associated anemia | 285.22 |
| Aplastic anemia | 244.9  284.01  284.89  284.9 |
| Sideroblastic anemia | 285.0  238.72  238.73 |
| Myelophthisic anemia | 284.2 |
| Pyridoxine-responsive anemia | 285.0 |
| G6PD deficiency | 282.2 |
| Pyruvate Kinase Deficiency | 282.3 |
| Pancytopenia | 284.1 |
| Fanconi anemia | 284.09 |
| Acquired and congenital pure red cell aplasia | 284.81  284.01 |
| Thrombotic thrombocytopenic purpura | 446.6 |
| Cold agglutinins | 283.0 |
| Mixed Cryoglobulinemia | 273.2 |
| Spleen Disorders (splenectomy, asplenia, splenomegaly, and hypersplenism) | 41.43  41.5  285.8  289.4  289.50  289.51  289.52  289.53  759.0  789.2 |
| Lead/ arsenic poisoning | 961.1  984.0  984.1  984.8  984.9  985.1 |
| Methanol poisoning | 980.1  987.8 |
| Sickle cell anemia | 282.5  282.60  282.62  282.63  282.64  282.68  282.69 |
| Thalassemias | 282.41  282.42  282.49 |
| Hereditary spherocytosis | 282.0  282.5  282.7 |
| Hereditary elliptocytosis | 282.1 |
| Hemoglobin C disease | 282.63  282.64 |
| Hemoglobin H disease | 282.49 |
| Paroxysmal nocturnal hemoglobinuria | 283.2 |
| ***S1.2. Hematologic Malignancies*** | |
| Leukemia | 200.x*  201.x  202.x  204.x  205.x  206.x  207.x  208.x |
| Multiple myeloma | 203.x |
| Polycythemia vera  (including secondary polycythemia) | 238.4  289.0 |
| Waldenstrom macroglobulinemia  Other paraproteinemia | 273.0  273.1  273.2  273.3  273.8  273.9 |
| ***S1.3. Gastrointestinal and Hepatic Disorders*** | |
| Cirrhosis | 571.x |
| Malabsorption disorders (eg, steatorrhea, celiac disease, and tropical sprue) | 579.0  579.1  579.2  579.4  579.8  579.9 |
| Crohn’s disease | 555.x |
| Ulcerative colitis | 556.x |
| ***S1.4. Autoimmune/Connective Tissue Disorders*** | |
| Ankylosing spondylitis | 720.x |
| Behçet disease | 136.1  711.2 |
| Buerger disease (thromboangiitis obliterans) | 443.1 |
| CREST | 710.1 |
| Essential cryoglobulinemic vasculitis | 273.2 |
| Felty syndrome | 714.1 |
| Henoch-Schönlein purpura | 287.0 |
| Polymyalgia rheumatica | 725 |
| Polymyositis/dermatomyositis | 710.3  710.4  359.7x |
| Rheumatoid arthritis | 714.x |
| Sarcoidosis | 135 |
| Scleroderma | 710.1 |
| Sjögren syndrome | 710.2 |
| Systemic Lupus erythematosus | 710.0 |
| Vasculitis (Churg-Strauss syndrome, cutaneous leukocytoclastic vasculitis, Goodpasture syndrome, hypersensitivity angiitis, Kawasaki disease, polyarteritis nodosa, Takayasu’s disease, temporal arteritis, and Wegener granulomatosis) | 446.x  447.6 |
| ***S1.5. Renal Disorders*** | |
| Chronic kidney disease (moderate and severe) | 585.3  585.4  585.5  585.6  585.9  788.9 |
| Glomerulonephritis (proliferative, membranoproliferative, crescentic) | 580.x  581.0  581.1  581.2  582.x |
| ***S1.6. Infectious Diseases*** | |
| Bacterial endocarditis | 421.x |
| Bronchiectasis | 494.x |
| Lung abscess | 513.x |
| Osteomyelitis | 730.x |
| Tuberculosis | 010.x–0.18.x |
