# Supplementary material for: A Genome-Wide Association Study of Red Blood Cell Traits Using the Electronic Medical Record
Source: PLoS One. 2010 Sep 28;5(9):e13011. doi: 10.1371/journal.pone.0013011 (PMC2946914; doi:10.1371/journal.pone.0013011)
Supplement: Table S2 — ICD-9 and CPT-4 procedural codes indicating bone marrow and/or solid organ transplantation. (0.05 MB DOC) [file pone.0013011.s006.doc]

| **Table S2.** ICD-9 and CPT-4 procedural codes indicating bone marrow and/or solid organ transplantation | |
| --- | --- |
| **Procedure** | **ICD-9 Code** |
| - Bone marrow transplant  Allogeneic | 41.00  41.03 |
| - Allogenic stem cell transplant | 41.05 |
| - Combined heart-lung transplant | 33.6 |
| - Allotransplantation of langerhans islets cells | 52.85 |
| - Kidney transplant NEC | 55.69 |
| - Liver transplant | 50.59  50.51 |
| - Lung transplant (single or bilateral) | 33.50 |
| - Pancreas transplant  Heterotransplant | 52.80  52.83 |
| - Transplant of islets of Langerhans (cells) | 52.86 |
| **Procedure** | **CPT-4 Code** |
| - Lung transplant, single, without cardiopulmonary bypass  - With cardiopulmonary bypass | 32851  32852 |
| - Double lung transplant, without cardiopulmonary bypass  - With cardiopulmonary bypass | 32853  32854 |
| - Heart-lung transplant with recipient cardiectomy-pneumonectomy | 33935 |
| - Heart transplant, with or without recipient cardiectomy | 33945 |
| - Renal allotransplantation, implantation of graft; without recipient nephrectomy  - With recipient nephrectomy | 50360  50365 |
| - Liver allotransplantation; orthotopic, partial or whole, from cadaver or living donor, any age  - Heterotopic, partial or whole, from cadaver or living donor, any age | 47135  47136 |
| - Transplantation of pancreatic allograft | 48554 |
| - Bone marrow or blood-derived peripheral stem cell transplantation; allogenic | 38240 |

*This represents all secondary ICD-9 CM codes under the main code.
