# Supplementary material for: A Genome-Wide Association Study of Red Blood Cell Traits Using the Electronic Medical Record
Source: PLoS One. 2010 Sep 28;5(9):e13011. doi: 10.1371/journal.pone.0013011 (PMC2946914; doi:10.1371/journal.pone.0013011)
Supplement: Table S3 — CPT-4 codes indicating medications. (0.04 MB DOC) [file pone.0013011.s007.doc]

| **Table S3.** CPT-4 codes indicating medications | |
| --- | --- |
| **Description of the Chemotherapeutic Procedure** | **CPT-4 Code** |
| - Chemotherapy administration, subcutaneous or intramuscular; non-hormonal anti-neoplastic  - Hormonal anti-neoplastic | 96401  96402 |
| - Chemotherapy administration; intralesional, up to and including 7 lesions  - Intralesional more than 7 lesions  - Intravenous, push technique, single or initial substance/ drug  - Intravenous, push technique, each additional substance/ drug | 96405  96406  96409  96411 |
| -Chemotherapy administration, intravenous infusion tenchnique, up to 1 hour, single or initial drug/ substance  - Same as above for each additional hour | 96413  96415 |
| -Initiation of prolonged chemotherapy infusion (>8 hrs)  - For each additional sequential infusion | 96416  96417 |
| - Chemotherapy administration, intra-arterial; push tech  - Infusion tech, up to 1 hr  - Infusion tech, each additional hr  - Infusion tech, initiation of prolonged infusion | 96420  96422  96423  96425 |
| - Chemotherapy administration into pleural cavity | 96440 |
| - Chemotherapy administration into peritoneal cavity | 96445 |
| - Chemotherapy administration into CNS (intrathecal) | 96450 |
| - Chemotherapy injection, subarachnoid or intraventricular via subcutaneous reservoir | 96542 |
| - Unlisted chemotherapy procedure | 96549 |
