# Supplementary material for: A Genome-Wide Association Study of Red Blood Cell Traits Using the Electronic Medical Record
Source: PLoS One. 2010 Sep 28;5(9):e13011. doi: 10.1371/journal.pone.0013011 (PMC2946914; doi:10.1371/journal.pone.0013011)
Supplement: Table S4 — Generic and brand names of commonly used oral chemotherapeutic and immunosuppressive medications. (0.05 MB DOC) [file pone.0013011.s008.doc]

| **Table S4.** Generic and brand names of commonly used oral chemotherapeutic and immunosuppressive medications |
| --- |
| - Methotrexate [MTX, amethopterin, Rheumatrex, Trexall, Folex PFS, Mexate] |
| - Azathioprine [Imuran, Azasan] |
| - 6-Mercaptopurine (6MP) [Purinethol, mercaptopurinum] |
| - Cyclophosphamide [Cytoxan, Neosar, Clafen] |
| - Hydroxyurea (Hydroxycarbamide) [Droxia, Hydrea] |
| - Imatinib [Gleevec] |
| - Dasatinib [Sprycel] |
| - Nilotinib [Tasigna] |
| - Busulfan [Busulfex, Myleran, Mitosan] |
| - Etoposide [VePesid, Toposar] |
| - Lomustine [Ceenu] |
| - Thioguanine (Tioguanine) [Tabloid, 6TG] |
| - Lenalidomide [Revlimid] |
| - Chlorambucil [Leukeran, linfolizin, amboclorin, ambochlorin] |
| - Melphalan [Alkeran] |
| - Trofosfamide [Ixoten] |
| - Mycophenolate mofetil (Mycophenolic acid) [CellCept] |
| - Capecitabine [Xeloda] |
| - Tegafur [Florafur, Fluorofur] |
| - Carmofur [HCFU] |
| - Cyclosporine [Restasis, Sandimmune, Neoral, SangCya] |
| - Tacrolimus [Prograf, Protopic] |
