# Supplementary material for: A Genome-Wide Association Study of Red Blood Cell Traits Using the Electronic Medical Record
Source: PLoS One. 2010 Sep 28;5(9):e13011. doi: 10.1371/journal.pone.0013011 (PMC2946914; doi:10.1371/journal.pone.0013011)
Supplement: Table S5 — CPT-4 codes indicating anesthesia codes for surgeries that are likely to be associated with major blood loss and post-operative anemia. (0.09 MB DOC) [file pone.0013011.s009.doc]

| **Table S5.** CPT-4 codes indicating anesthesia codes for surgeries that are likely to be associated with major blood loss and post-operative anemia | | |
| --- | --- | --- |
| **Surgery for which anesthesia was administered** | **CPT-4 Code** | |
| ***S5.1. Head*** | | |
| -Anesthesia for procedures on nose and accessory sinuses  Radical surgery | 00162 | |
| -Excision of retropharyngeal tumor  Radical surgery | 00174  00176 | |
| -Anesthesia for procedures on facial bones or skull; NOS  Radical surgery | 00190  00192 | |
| -Anesthesia for intracranial procedures; NOS  -Craniotomy or craniectomy for evacuation of hematoma  -Burr holes, including ventriculography  -Cranioplasty or elevation of depressed skull fracture, extradural (simple or complicated)  -Vascular procedures | 00210  00211  00214  00215  00216 | |
| ***S5.2. Neck*** | | |
| -Anesthesia for all procedures on the integumentary system, muscles and nerves of head, neck, and posterior trunk, NOS | 00300 | |
| -Anesthesia for all procedures on esophagus, thyroid, larynx, trachea and lymphatic system of neck; NOS (> 1 years old) | 00320 | |
| -Anesthesia for procedures on major vessels of neck; NOS | 00350 | |
| ***S5.3. Thorax (Chest Wall and Shoulder Girdle)*** | | |
| -Anesthesia for procedures on the integumentary system on the extremities, anterior trunk and perineum?; NOS | 00400 | |
| -Radical or modified radical procedures on breast | 00404 | |
| -Radical or modified radical procedures on breast with internal mammary node dissection | 00406 | |
| -Radical surgery of the thorax | 00452 | |
| -Thoracoplasty (any type) | 00472 | |
| -Radical procedures (eg. pectus excavatum) | 00474 | |
| ***S5.4. Intrathoracic Surgeries*** | | |
| -Anesthesia for all procedures on esophagus | 00500 | |
| -Anesthesia for tracheobronchial reconstruction | 00539 | |
| -Anesthesia for thoracotomy procedures involving lungs, pleura, diaphragm, and mediastinum (including surgical thoracoscopy); NOS  -As above utilizing lung ventilation  -Decortications  -Pulmonary resection with Thoracoplasty  -Intrathoracic procedures on the trachea and bronchi | 00540  00541  00542  00546  00548 | |
| -Anesthesia for procedures on heart, pericardial sac, and great vessels of chest; without pump oxygenator  -As above with pump oxygenator (>1 yo) for all non-coronary bypass procedures or for re-operation for CABG  -With pump oxygenator with hypothermic circulatory arrest | 00560  00562  00563 | |
| -Anesthesia for direct CABG without pump oxygenator  -With pump oxygenator | 00566  00567 | |
| -Anesthesia for heart transplant or heart/ lung transplant | 00580 | |
| ***S5.5. Spine and Spinal Cord*** | | |
| -Anesthesia for procedures on thoracic spine and cord, via an anterior transthoracic approach; utilizing lung ventilation | 00626 | |
| -Anesthesia for procedures in the lumbar region; NOS | 00630 | |
| -Anesthesia for extensive spinal cord procedures (eg, spinal instrumentation or vascular procedures) | 00670 | |
| ***S5.6. Upper Abdomen*** | | |
| -Anesthesia for transabdominal repair of diaphragmatic hernia | 00756 | |
| -Anesthesia for all procedures on major abdominal blood vessels | 00770 | |
| -Anesthesia for intraperitoneal procedures in upper abdomen including laparoscopy; NOS | 00790 | |
| -Partial hepatectomy or management of liver hemorrhage (excluding liver biopsy) | 00792 | |
| -Pancreatectomy, partial or total | 00794 | |
| -Liver transplant (recipient) | 00796 | |
| -Gastric restrictive procedure for obesity | 00797 | |
| ***S5.7. Lower Abdomen*** | | |
| -Anesthesia for intraperitoneal procedures in lower abdomen including laparoscopy; NOS  -Abdominoperineal resection  -Radical hysterectomy  -Pelvic exenteration | 00840  00844  00846  00848 | |
| -Anesthesia for extraperitoneal procedures in lower abdomen, including urinary tract; NOS  -Renal procedures including upper one-third of ureter, or donor nephrectomy  -Total cystectomy  -Radical prostatectomy (suprapubic/ retropubic)  -Adrenalectomy | 00860  00862  00864  00865  00866 | |
| -Renal transplant (recipient) | 00868 | |
| -Anesthesia for procedures on major lower abdominal vessels; NOS | 00880 | |
| ***S5.8. Perineum*** | | |
| -Radical perineal procedure  -Perineal prostatectomy  -Post-transurethral resection bleeding | | 00904  00908  00916 |
| -Anesthesia for vaginal hysterectomy | | 00944 |
| ***S5.9. Pelvis (Except Hip)*** | | |
| -Anesthesia for interpelviabdominal (hindquarter) amputation | | 01140 |
| -Anesthesia for radical procedures for tumor of pelvis, except hindquarter amputation | | 01150 |
| -Anesthesia for open repair of fracture disruption of pelvis or column fracture involving acetabulum | | 01173 |
| ***S5.10. Upper Leg (Except Knee)*** | | |
| -Anesthesia for open procedures involving hip jopint; NOS  -Hip disarticulation  -Total hip arthroplasty  -Revision of total hip arthroplasty | | 01210  01212  01214  01215 |
| -Anesthesia for open procedures involving upper 2 thirds of femur; NOS  -Amputation  -Radical resection | | 01230  01232  01234 |
| -Anesthesia for all procedures involving veins of upper leg, including exploration | | 01260 |
| -Anesthesia for procedures involving arteries of upper leg, including bypass graft; NOS | | 01270 |
| ***S5.11. Knee and Popliteal Area*** | | |
| -Anesthesia for all open procedures on lower one third of femur | | 01360 |
| -Anesthesia for all open procedures on upper ends of tibia, fibula, and/or patella | | 01392 |
| -Total knee arthroplasty  -Disarticulation at knee | | 01402  01404 |
| -Anesthesia for procedures on arteries on knee and Popliteal area; NOS  -Popliteal thrombendarterectomy, with or without patch graft  -Popliteal excision and graft or repair for occlusion or aneurysm | | 01440  01442  01444 |
| ***S5.12. Lower Leg (Below Knee, Includes Ankle and Foot)*** | | |
| -Radical resection (including below knee amputation)  -Osteotomy or ostepoplasty of tibia and/or fibula  -Total ankle replacement | | 01482  01484  01486 |
| -Anesthesia for procedures on arteries of lower leg, including bypass graft; NOS | | 01500 |
| ***S5.13. Shoulder and Axilla*** | | |
| -Shoulder disarticulation  -Interthoracoscapular (forequarter) amputation  -Total shoulder replacement | | 01634  01636  01638 |
| -Anesthesia for procedures on arteries of shoulder and axilla; NOS  -Axillary-brachial aneurysm  -Bypass graft  -Axillary-femoral bypass graft | | 01650  01652  01654  01656 |
| ***S5.14. Upper Arm and Elbow*** | | |
| -Osteotomy of humerus  -Radical procedures  -Excision of cyst or tumor of the humerus  -Total elbow replacement | | 01742  01756  01758  01760 |
| -Anesthesia for procedures on arteries of upper arm and elbow; NOS | | 01770 |
